# The Timing of the Cognitive Cycle

**DOI:** 10.1371/journal.pone.0014803

**Published:** 2011-04-25

**Authors:** Tamas Madl, Bernard J. Baars, Stan Franklin

**Affiliations:** 1 Department of Philosophy (Cognitive Science), University of Vienna, Vienna, Austria; 2 The Neurosciences Institute, San Diego, California, United States of America; 3 Institute for Intelligent Systems, The University of Memphis, Memphis, Tennessee, United States of America; University College London, United Kingdom

## Abstract

We propose that human cognition consists of cascading cycles of recurring brain events. Each cognitive cycle senses the current situation, interprets it with reference to ongoing goals, and then selects an internal or external action in response. While most aspects of the cognitive cycle are unconscious, each cycle also yields a momentary “ignition” of conscious broadcasting. Neuroscientists have independently proposed ideas similar to the cognitive cycle, the fundamental hypothesis of the LIDA model of cognition. High-level cognition, such as deliberation, planning, etc., is typically enabled by multiple cognitive cycles. In this paper we describe a timing model LIDA's cognitive cycle. Based on empirical and simulation data we propose that an initial phase of perception (stimulus recognition) occurs 80–100 ms from stimulus onset under optimal conditions. It is followed by a conscious episode (broadcast) 200–280 ms after stimulus onset, and an action selection phase 60–110 ms from the start of the conscious phase. One cognitive cycle would therefore take 260–390 ms. The LIDA timing model is consistent with brain evidence indicating a fundamental role for a theta-gamma wave, spreading forward from sensory cortices to rostral corticothalamic regions. This posteriofrontal theta-gamma wave may be experienced as a conscious perceptual event starting at 200–280 ms post stimulus. The action selection component of the cycle is proposed to involve frontal, striatal and cerebellar regions. Thus the cycle is inherently recurrent, as the anatomy of the thalamocortical system suggests. The LIDA model fits a large body of cognitive and neuroscientific evidence. Finally, we describe two LIDA-based software agents: the LIDA Reaction Time agent that simulates human performance in a simple reaction time task, and the LIDA Allport agent which models phenomenal simultaneity within timeframes comparable to human subjects. While there are many models of reaction time performance, these results fall naturally out of a biologically and computationally plausible cognitive architecture.

## Introduction

Cognitive science and cognitive neuroscience aim at understanding and explicating human cognition. The extraordinary complexity and interconnectivity of human cognitive processing [1], taken together with the intricate interactivity of these processes, cries out for the use of broad, comprehensive, integrated cognitive architectures [2],[3]. Such architectures have played an ongoing major role in the development of cognitive science [3]–[6]. Hypotheses from such comprehensive, integrated architectures serve to guide research in cognitive science and cognitive neuroscience. As does human cognition, each of these architectures performs via cyclic iteration of a collection of primary processes.

We humans are confronted with a world full of action choices. Using various cognitive processes, we have to decide what to do next and thus answer what can be seen as the only question there is: “What shall I do next?” (see Franklin's Action Selection paradigm [7]). In this way, every autonomous agent [8], be it human, animal, or artificial, must frequently sample (sense) its environment, process (make sense of) the input from such sampling, and select an appropriate response (action).

In the LIDA (Learning Intelligent Distribution Agent) cognitive architecture [8], with which we will be concerned here, the repeated cycle of perception, understanding and action selection is called a cognitive cycle. The same idea has been proposed in similar form in different fields by different authors, for example the action-perception cycles in neuroscience [8]–[12], the intentional arc [13], or the recognize-analyze-synthesize cycle in systems engineering [14].

The most important hypothesis put forth by LIDA is that *such cognitive cycles are the fundamental building blocks of all human cognition*: ‘*cognitive atoms’*. Complex cognitive tasks, such as non-routine problem solving, deliberation, volitional decision making, higher-level perception or imagination, can require many of these cycles, several of which can cascade as long as the seriality of consciousness is preserved [8], [15], [16]. Within each cognitive cycle a number of modules and processes operate, varying with the current situation or task. The LIDA cognitive cycle is consistent with many neuroscientific findings, as can be seen from the evidence presented in this paper. If human cognition consists of these cognitive cycles, as the empirical evidence strongly suggests [17]–[24], it is imperative to find out as many details about the operation of their modules and processes as possible. Our description of the internal and external timings of such cognitive cycles is an attempt to contribute to this goal.

In this paper we propose a timing model of the cognitive processes humans employ from sensing to action selection, based on recent neuroscientific findings. We will categorize such processes into different stages within the scope of the LIDA cognitive cycle, use recent neuroscientific findings to correlate them with relevant brain areas, and suggest ranges of how long the processing in these brain areas could take (see Results section). We will also compare our timing model with a few other influential cognitive models (Section 3). Finally, we will introduce two autonomous software agents based on the computational LIDA framework [8]. The first agent performs simple reaction time experiments and produces actions in times similar to human subjects; and the second models phenomenal simultaneity within timeframes comparable to human subjects. Both agents use cognitive processes comparable to humans.

### LIDA and Consciousness

The LIDA model is a comprehensive, cognitive model that, with its computational architecture, covers a large portion of human cognition. Based primarily on global workspace theory [25], the model implements and fleshes out central ideas from a number of psychological and neuropsychological theories including situated (embodied) cognition [26], [27], perceptual symbol systems [28], working memory [29], memory by affordances [30], long-term working memory [31], transient episodic memory [32], and Sloman's H-CogAff cognitive architecture [33].

LIDA's cognitive cycle consists of multiple modules, which can be partitioned into the three stages of the perception-understanding-action cycle. The computational LIDA framework has been almost completely implemented, and serves as a basis for the two computational agents demonstrating the timings of the cognitive cycle (see Results section).

As mentioned above, the LIDA model is based on the global workspace theory of consciousness [25], which suggests the existence of a fleeting memory capacity that enables access between brain functions that are otherwise separate. The global workspace theory (GWT) can be thought of as “… a theater of mental functioning. Consciousness in this metaphor resembles a bright spot on the stage of immediate memory, directed there by a spotlight of attention under executive guidance. Only the bright spot is conscious, while the rest of the theater is dark and unconscious” [34]. In case of sensory consciousness, the stage corresponds to the sensory projection areas of the cortex, its activation coming either from senses or from internal sources. After a conscious sensory content is established, it is distributed to a decentralized “audience” of expert networks sitting in the darkened theater. Thus, the primary functional purpose of consciousness is to integrate, provide access, and coordinate the functioning of very large numbers of specialized networks that otherwise operate autonomously. In the neuroscientific study of consciousness, this idea of consciousness having an integrative function has proven very useful, and is supported by much recent evidence [34]–[36] (see also the Results section).

**Figure 1:**
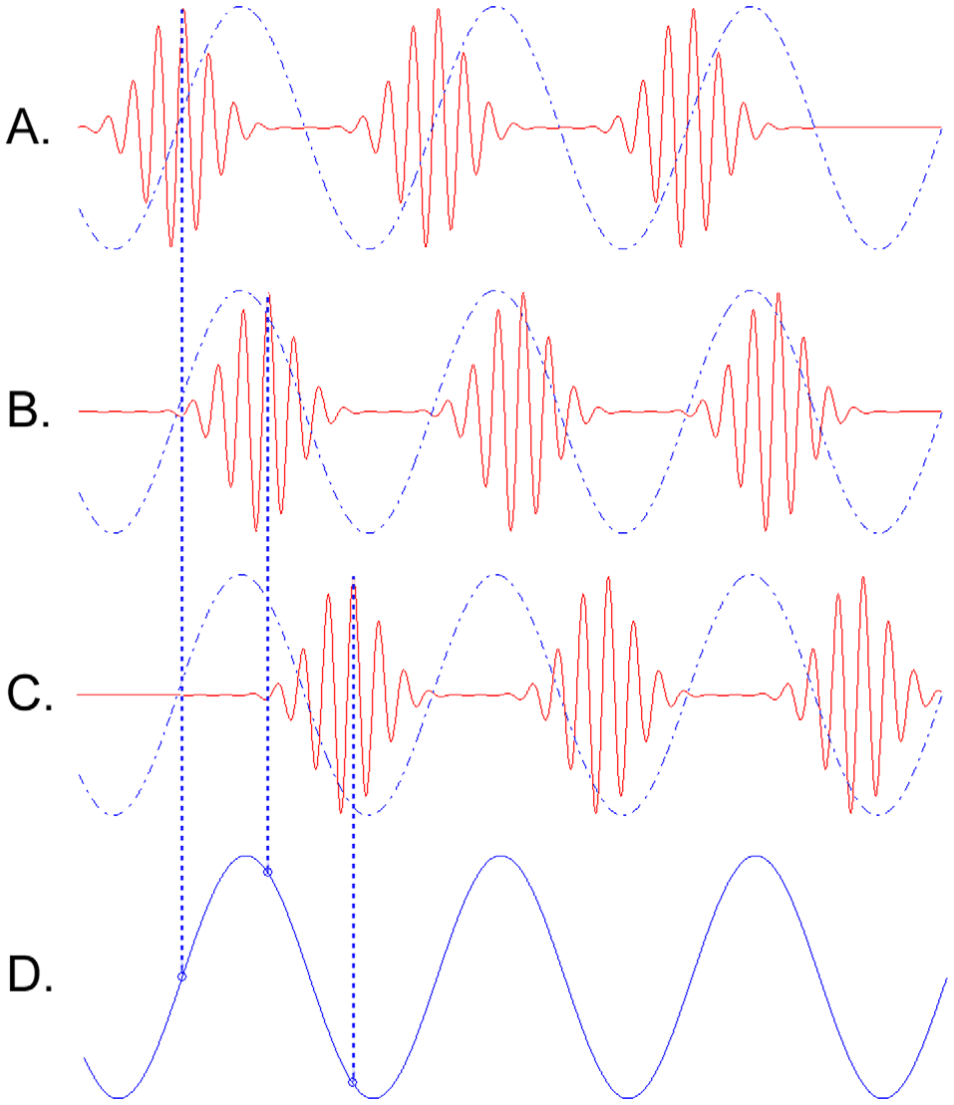
Theta-gamma coupling. Three gamma cycles are sequentially “embedded” in a theta cycle. (A), (B), and (C) depict the temporal activity pattern of three different neuronal assemblies oscillating in the gamma range. Each is phase-locked to the underlying theta rhythm with a different phase offset, as indicated by the dashed lines. This type of coupling is known as phase-amplitude coupling, because the amplitude modulation of each gamma pattern is locked to a particular phase of the theta pattern (S).

In LIDA, every cognitive cycle can have only a single conscious “frame” (content) at a time, a hypothesis compatible with recent neuroscientific publications which view consciousness as large-scale phase synchronization of neuronal activity [37]–[40]. In this view, the complex rearrangement of neural populations across widespread and diverse cortical regions, which is required for consciousness, is accomplished by oscillatory dynamics; specifically, by theta-gamma coupling between the neural populations (see Figure 1 - *from* [*38*] *with permission*).

**Figure 2:**
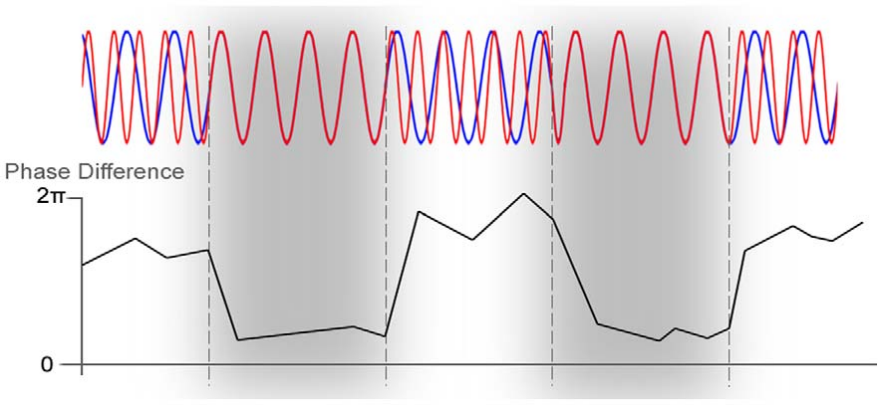
Phase synchrony between two oscillations. The upper part shows two oscillations (in red and blue), and the lower part their phase-differences. In the two gray areas framed by dotted lines the oscillations are highly phase synchronous and the phase differences are low. Such phase-synchrony in the gamma band has been proposed to be responsible for perceptual binding (for example, cortical columns representing the same object are gamma synchronized).

Performing cognitive tasks modulates oscillatory brain activity in various frequency bands, including both the theta (4–7 Hz) and gamma (30–150 Hz) bands. Gamma-band phase synchrony (Figure 2) has been associated with perceptual binding and awareness. Numerous studies have observed the occurrence of gamma activity coherence with perceptual [41], [42] as well as long-term [43] and working-memory-related [39] object representations. Synchronized gamma-band oscillatory activity has also been shown to play an important role in the coding of short-term memory information [24], [44], [45]. Moreover, modulation of gamma activity has been demonstrated in attentional selection [46]-[48], and phase-locked gamma synchrony between ascending and descending systems in a sensorimotor task [39]. Many of these studies have observed that activity across different cortical columns representing the percept of an object is gamma synchronized (e.g. [42]). Thus, the neuronal ensembles responsible for various cognitive processes involved in the processing of a percept, taking place during a cognitive cycle, operate at and are integrated by an internal oscillation frequency in the gamma band.

The construction of such gamma-synchronous neural ensembles has been claimed to be governed by theta-rhythms [37], [49]. This might be the integration mechanism required for consciousness: in this view, consciousness emerges from large-scale functional integration of these gamma-synchronous ensembles that form and dissolve at the theta frequency band [37].

Only one perceptual experience can be contained in a single phase of theta-modulated gamma-synchrony [37], consistently with the attentional blink ([50], see also Results section) and other studies of perceptual synchrony [51]. This indicates that these phases of synchrony define discrete ‘frames’ of consciousness, which, in the LIDA model, correspond to cognitive cycles [16], [38]. An approximate lower time limit for a single cognitive cycle can already be deduced from this hypothesis. Since each cycle is concerned with a single conscious content, and a new conscious content requires theta-gamma synchronization, conscious processing in the cognitive cycles has to occur at theta rates (4–7 Hz). Therefore cognitive cycles have to take at least 140–250 ms. However, since cognitive cycles can cascade as long as they preserve the seriality of consciousness, they could take longer than that (see Results section).

An important hypothesis of the LIDA model is the discreteness of consciousness. Humans can only have a single conscious content at a time, and there are short breaks between these periods of consciousness. In the words of Franklin et al. [8], “conscious events occur as a sequence of discrete, coherent episodes separated by quite short periods of no conscious content” (see also [52]) - similar to the frames of a movie, the ‘frames’ of consciousness are discrete but are experienced as being continuous (although this analogy is not entirely accurate).

This view is consistent with the idea of consciousness emerging from theta-gamma coupling. Gamma-oscillatory neural ensembles are synchronized as well as desynchronized at theta rates. The transient periods of desynchronization, also called phase scattering, reflect unconscious processing in the brain, thus “ending each ‘frame’ of [conscious] perceptual experience” [37]. These periods of desynchronization have also been observed, and pointed out, to play a role in the transition from one cognitive content to another by [51],[53]–[56]. (For more neuroscientific results about consciousness see the Results and Discussion section below). In psychology, Stroud [57] was one of the first authors to propose the idea of discrete frames or ‘moments’ underlying consciousness. His ‘Discrete Moment Hypothesis’ included two important underlying assumptions: a) a complete loss of time-order information within one conscious ‘moment’, and b) a distinct and non-overlapping set of percepts for each ‘moment’. This strict view of discrete consciousness has been regarded with some skepticism. Allport [58], for instance, has conducted experiments on phenomenal simultaneity, which seem to contradict the Discrete Moment Hypothesis – they are, however, compatible with LIDAs consciousness model, as can be seen from the Results section, in which we replicated the data from Allport's experiment using a LIDA-based agent.

**Figure 3:**
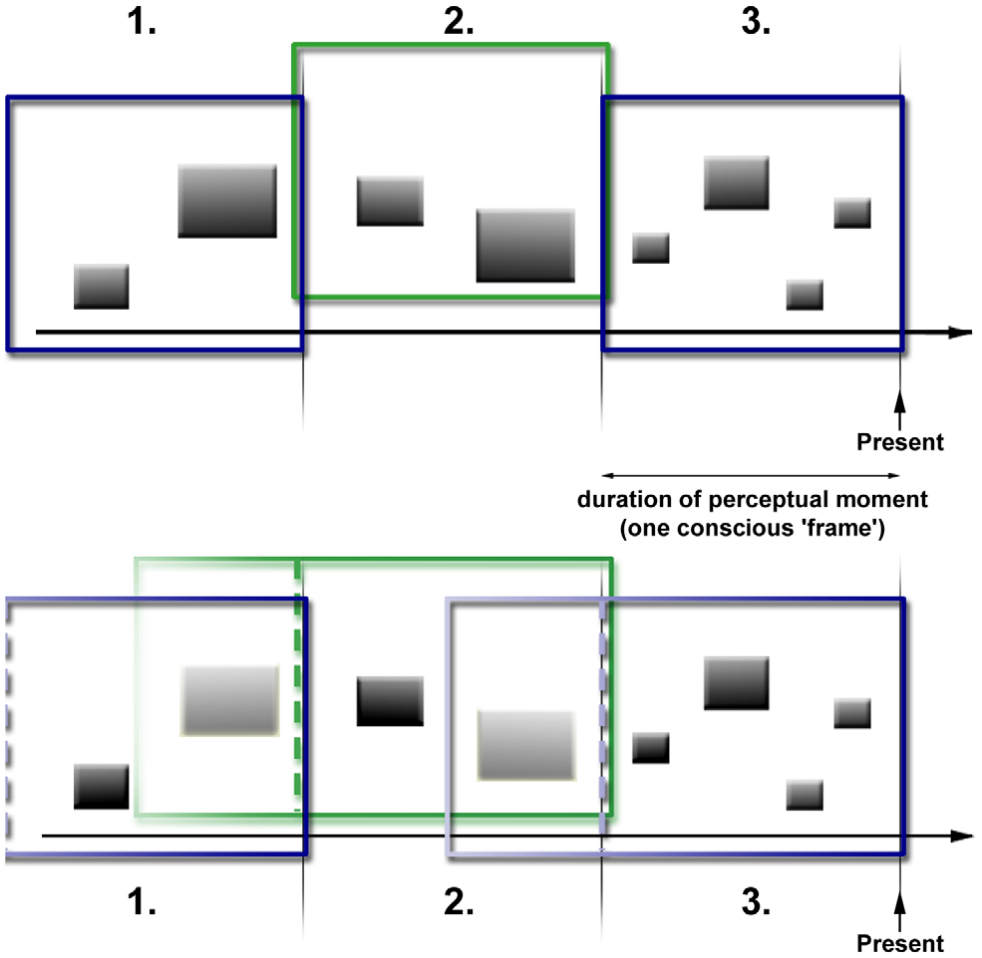
Schematic comparison of the Discrete Moment Hypothesis (top) and LIDA's discrete consciousness hypothesis (bottom). The colored frames represent the temporal constraints of a perceptual moment or conscious ‘frame’, and the black rectangles symbolize incoming percepts. In LIDA, important percepts from previous conscious ‘frames’ can remain conscious (rectangles left of the dashed lines in the coloured frames in the bottom picture).

In the LIDA model, single conscious episodes are discrete but, contrary to Stroud's [57] view, not necessarily distinct – a current conscious ‘moment’ can contain percepts from a previous moment. Whether or not an older percept remains conscious depends on how long in the past it has been perceived, and on attentional modulation – percepts that are subjectively important and attended to can persist longer in consciousness. To improve our earlier movie analogy, the ‘frames’ of consciousness in the LIDA model could be compared to a movie shown on a phosphor-based electronic display (CRT): although the frames are discrete, new images on the screen contain past information (see Figure 3). As we will see in the Results section, this approach resolves the empirical contradictions of the Discrete Moment Hypothesis.

Since our timing model was largely derived from neuroscientific experiments, some tools and techniques these experiments might use, and the reasons we preferred to use the results of some experiments over others, should be described.

Electroencephalography (EEG) records electrical activity from neural field generators using several electrodes placed on the scalp surface. Recent research concentrates on aspects of this electrical activity time-locked to events, i.e. event-related potentials (ERP), which occur in preparation of or in response to discrete (internal or external) events. We have used EEG experimental results because EEG has great temporal resolution (on the order of milliseconds), and a large number of EEG results are available. Disadvantages of EEG are its low spatial resolution (typically 2–3 cm in surface tangential directions) and the fact that it only measures synaptic activity from superficial cortical layers [59].

Transcranial magnetic stimulation (TMS) experiments involve stimulating the brain using induced electric currents, which trigger action potentials in the neurons in the current field, disrupting ongoing brain activity (causing temporary “virtual lesions”). We also used TMS experiments because TMS resolutions are very good (temporal resolution on the order of milliseconds, spatial resolution on the order of a few millimeters, depending on the coil shape). Disadvantages of TMS are the impossibility to determine exactly how much area is affected by these induced currents. Also, TMS cannot stimulate regions deeper than the cortex without stimulating the cortex.

The most exact technique measuring brain activity is using depth electrode and subdural grid recordings. Depth electrode recordings are mostly performed on animals and clinical patients. Subdural grid recordings (also called electrocorticograms or ECoG), involving the placing of electrodes directly on the brain surface, are less invasive and have spatial resolution somewhere between depth electrodes and EEG. These techniques provide the most exact and reliable data, but they require surgery and cannot be used in healthy humans [39].

The reason we have not used experiments relying solely on functional magnetic resonance imaging (fMRI) data is that this technique measures blood oxygen levels, and it takes several minutes for the bloodstream in active brain areas to become oxygenated [60], which is well outside our time scale.

A more complete and detailed review of non-invasive brain imaging techniques can be found in [61].

### The LIDA Cognitive Cycle

Autonomous agents [62] cope with their changing environment by their continuous, cyclic chores of ‘perceive-understand-act’. LIDA's cognitive cycle [8] is the cycle of refined cognitive processes (starting after sensation and ending with action) that bring about the appropriate action for specific situation. As Franklin and Baars [16] put it ‘A cognitive cycle can be thought of as a moment of cognition - a cognitive moment; higher-level cognitive processes are composed of many of these cognitive cycles, each a cognitive atom.’ This metaphor is to say that the steps in a cognitive cycle correspond to the various sub-atomic particles in an atom.

**Figure 4:**
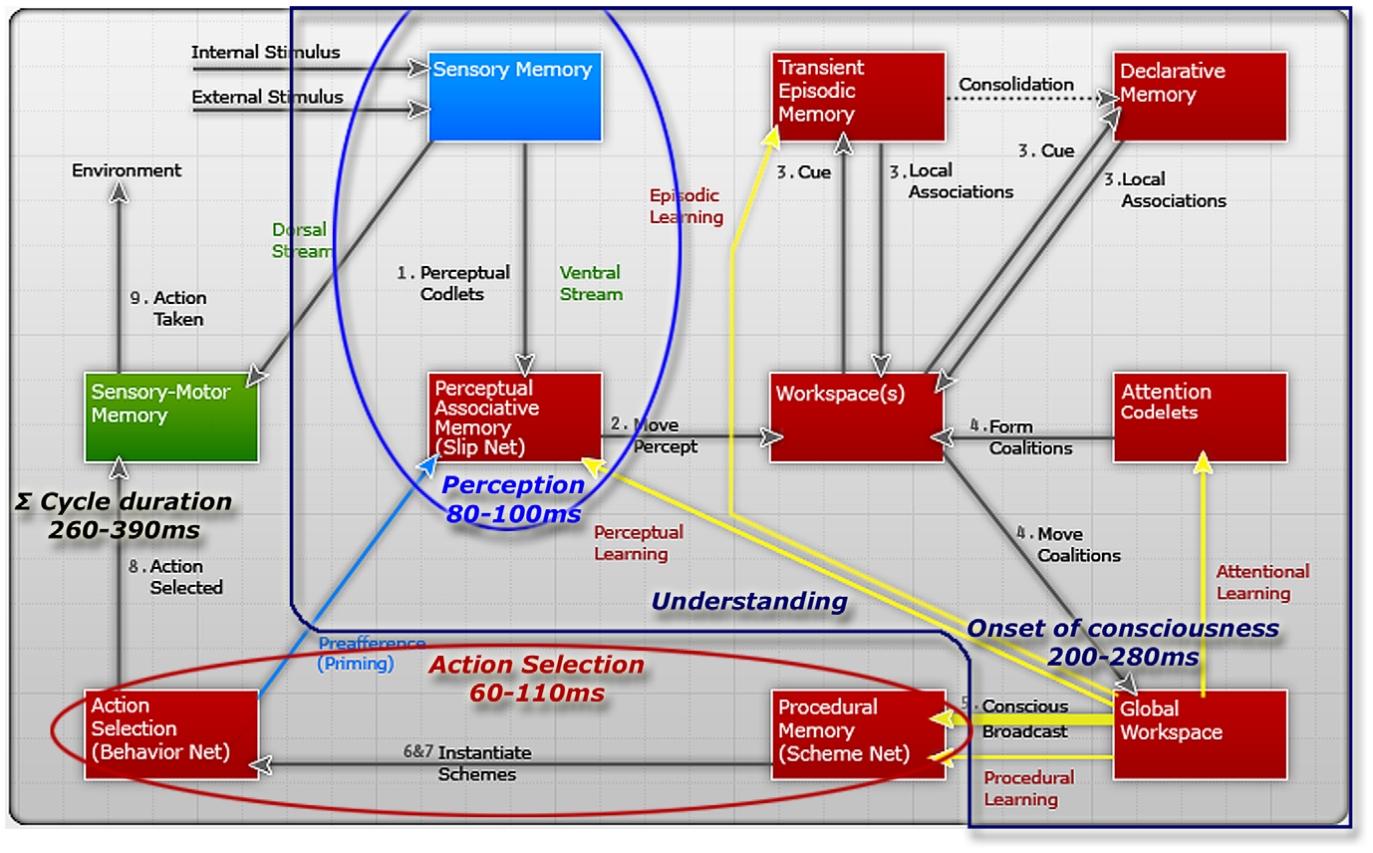
The LIDA cognitive cycle, and the durations of the perception, understanding and action phases.

Since the LIDA architecture is composed of several specialized mechanisms, a continual process that causes the functional interaction among the various components is essential. The cognitive cycle as such is an iterative, cyclical, continually active process that brings about the interplay among the various components of the architecture. The steps of cognitive cycle are shown in Figure 4 (*Modified from* [*63*]) and will be described below. It is important to point out the asynchrony of the LIDA cognitive cycle. Cycles can cascade as long as they preserve the seriality of consciousness. Furthermore, the components of the cognitive cycle described below should not be seen as serial stages of information processing. The components operate asynchronously - although coordinated, each component has its own internal mechanism and agenda. Components receiving inputs from others are not triggered by those inputs, but rather run continuously at their specified frequencies of operation (See Methods section).

During each cognitive cycle the LIDA agent, be it human, animal or artificial, first senses its environment and tries to recognize familiar objects, individuals, etc (perception phase). It then associates percepts with memories and other percepts and decides what portion of this situation is most in need of attention (understanding phase). Broadcasting this portion (bringing it to consciousness) enables the agent to choose a number of actions applicable for the current situation and to select the action best serving its goals (action selection phase), and to finally execute the selected action. The cognitive cycle has the following components:

1) **Perception**. Sensory stimuli, external or internal, are received and interpreted by perception producing the beginnings of meaning.

2) **Percept to preconscious buffer**. The percept, including some of the data plus the meaning, as well as possible relational structures, is stored in the preconscious buffers of LIDA's working memory (workspace). Temporary structures are built.

3) **Local associations**. Using the incoming percept and the residual contents of working memory, including emotional content, as cues, local associations are automatically retrieved from transient episodic memory and from declarative memory, and stored in long-term working memory.

4) **Competition for consciousness**. Attention codelets view long-term working memory, and bring novel, relevant, urgent, or insistent events to consciousness.

5) **Conscious broadcast**. A coalition of codelets, typically an attention codelet and its covey of related informational content, gains access to the global workspace and has its content broadcast consciously. Thus consciousness solves the relevancy problem in recruiting resources.

6) **Recruitment of resources**. Relevant schemes in Procedural Memory respond to the conscious broadcast. These are typically schemes (underlain by behavior codelets) whose context is relevant to information in the conscious broadcast. Thus consciousness solves the relevancy problem in recruiting resources.

7) **Setting goal context hierarchy**. The recruited schemes use the contents of consciousness, including feelings/emotions, to instantiate new goal context hierarchies (copies of themselves) into the Action Selection system), bind their variables, and increase their activation. Other, environmental, conditions determine which of the earlier behaviors (goal contexts) also receive variable binding and/or additional activation.

8) **Action chosen**. The Action Selection module chooses a single behavior (scheme, goal context), from a just instantiated behavior stream or possibly from a previously active stream. Each selection of a behavior includes the generation of an expectation codelet (see the next step).

9) **Action taken**. The execution of a behavior (goal context) results in the behavior codelets performing their specialized tasks, having external or internal consequences, or both. LIDA is taking an action. The acting codelets also include at least one expectation codelet whose task it is to monitor the action, bringing to consciousness any failure in the expected results.

As shown in Figure 4, multiple learning mechanisms are initiated following the broadcast of conscious content. In the perceptual associative memory learning of new entities and associations, and the reinforcement of old ones occur, events are encoded in the Transient Episodic Memory, and new schemes may be learned and old schemes reinforced in Procedural Memory; in all of the learning processes, the conscious content determines what is to be learned. For more information about the LIDA model and its cognitive cycle see [8], [16].

## Results and Discussion

As mentioned above, cognition in autonomous agents [62], whether artificial, animal or human, can be thought of as consisting of repeated perception-understanding-action cycles. In these cycles, actions can be external (effecting changes in the environment) or internal (effecting changes in internal representations or processes). Similarly, perceptual information can come from external (from senses sensing the environment) or internal sources. Complex tasks may require many of these cycles before an external action can be taken.

**Figure 5:**
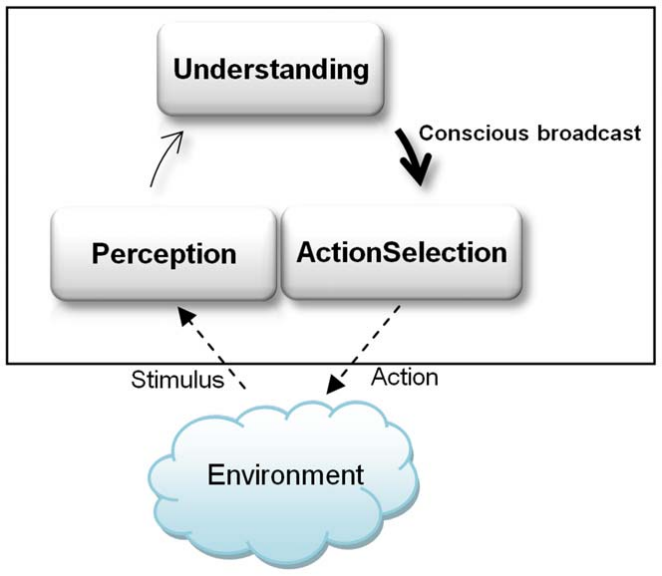
The three phases of the LIDA cognitive cycle. A stimulus comes in from the environment via the senses. The perception sub-process includes obtaining this data, detecting features, and recognizing objects, categories and events. The understanding sub-process includes making sense of the perceived information and selecting the most relevant, urgent or novel information, which is included in the conscious broadcast (the agent is only consciously aware of the contents of this broadcast). Finally, the action selection sub-process selects the action best serving the agent's goals, based on the conscious broadcast contents.

**Figure 6:**
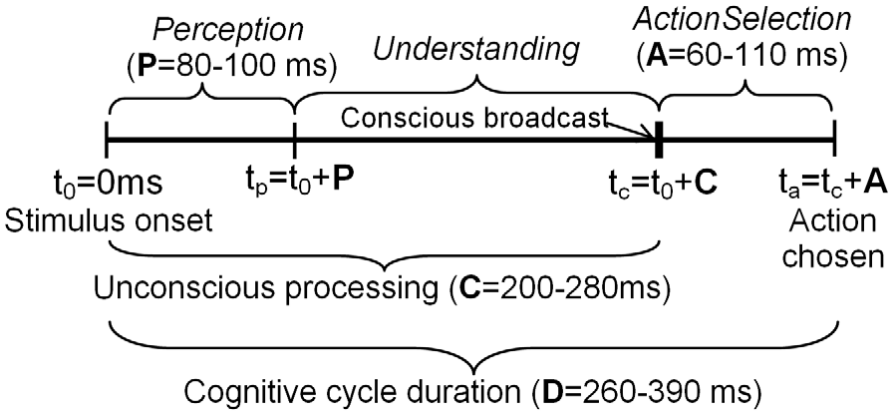
The timing of a single cognitive cycle. The perception sub-process is estimated to take P = 80–100 ms, the time until conscious processing C = 200–280 ms, the action selection sub-process A = 60–110 ms, and the entire cognitive cycle is hypothesized to take D = 260–390 ms.

Figure 5 shows such a cognitive cycle, including its three sub-processes. For the durations of these sub-processes, see Figure 6.

The understanding phase in this cognitive cycle is frequently called ‘cognition’ in other cognitive models (e.g. [64], [65]). In LIDA, the term ‘understanding’ is more appropriate because the integration of percepts, the building of associations (with memories and with other percepts) and assessments of subjective significance that take place during this phase all contribute to a representation or situational model (stored in temporary memory, the workspace) which is best described as the agents current understanding of its immediately perceived environment (see Introduction). In other cognitive models, such as ACT-R or EPIC, the cognition phase includes the matching, selection and execution of production rules [64], [65].

Figure 6 shows our hypothesized durations for the sub-processes of the cognitive cycle in humans. The next subsections will describe neural equivalents of these sub-processes and provide supporting evidence for the indicated durations. The indicated ranges should not be taken as precise and definite values; rather, they are working estimates derived from recent evidence.

It should be pointed out that the experiments on which these durations are based used very simple settings and stimuli, and in most cases, they did not involve memory recall. For tasks involving the use of memory, the time from stimulus presentation to action execution can be significantly longer than the times indicated here [66]. However, for most simple tasks, due to the large extent of consistency between these results and various psychological and neuroscientific experiments (see below), we believe that the indicated durations of these processes accurately reflect some of the temporal properties of human cognition.

### Perception

The perception process includes obtaining data from the environment via sensors, detecting features, and recognizing more abstract entities such as objects,events and categories.

In humans, perceptual information can come from different sensory modalities. The most researched and perhaps most complex modality (judging from the size of cortical areas associated with its processing) is visual perception [67].

Visual perception starts with an image of the environment on the photoreceptive cells of the retina, which produces neural impulses that are transmitted along the retinofugal projection to the visual cortex, which is located in the occipital lobe, where most of the processing of visual information takes place [67].

We have estimated the duration of the perception process in humans for simple tasks to be approximately in the range of P  = 80–100 ms (see Figure 6). For instance, an experiment by Liu et al. [68], performed using intracranial electrodes in epilepsy patients, has shown that object category information can be decoded from neural activity in the occipital lobe as early as 100 ms poststimulus. This is consistent with EEG experiments trying to temporally localize object-selective brain activity, most of which found that the P100 ERP component (90–115 ms post stimulus) is already associated with object information [69]. It is also consistent with the result of various studies of visual processing which have determined that a stimulus presentation time of 100 ms is sufficient for recognizing traits and properties [70], [71]. Finally, this duration was also indicated by TMS experiments investigating in which time range TMS interferences with the visual system can impede vision. Such experiments found that the range of greatest impairment was between 80 and 100 ms, and that TMS interference after 100 ms had little to no effect on visual perception [72], [73].

This perceptual duration seems to provide an appropriate upper limit for the perception process in general, since information from other modalities is processed in this range or even faster in the human brain. For example, auditory (and somatosensory) event related responses in the sensory cortices can commence in less than 50 ms [74], and the entire auditory neural representation can be built during the N1 stage in ∼100 ms [75], [76].

### Cognitive Processing and Consciousness

According to the LIDA model and GWT (see Introduction), a major functional role of consciousness is to distribute important perceptual information to different, specialized brain areas. (Novel Hypothesis 5 in [8]) It is possible to derive a way to measure the elapsed time between the sensing of a stimulus and its becoming a conscious event from this hypothesis. Unconscious processing of the stimulus appears to be more localized in sensory areas (e.g. the visual cortex for visual stimuli), meaning that these areas have the highest activity in the unconscious processing stage. Conscious processing can be said to start at the moment other brain areas, for example those involved in decision making/action selection (e.g. pre-frontal areas, see next section), become highly active – this information can be derived from fast brain imaging techniques.

There are experimental indications that this distribution of information, termed the conscious broadcast [16] commences about 200–280 ms post stimulus (Figure 6).

For substantiating the claim of when conscious processing starts, comparisons of conscious and non-conscious processing of the same stimulus are sometimes used. There are a number of such neuroscientific experiments that yield useful timing results from this point of view. Gaillard et al. [77] have conducted an intracranial iEEG experiment using a visual masking procedure, performing trials with and without conscious visibility of masked words (with and without showing a mask very shortly after presenting the word), concluding that conscious processing takes place 200–300 ms post stimulus. Other studies using EEG and also using a masked visual paradigm indicated conscious processing to commence at 270 ms [78], [79] (see also the survey about conscious and unconscious processing in [36]). An MEG study using a different visual paradigm (subjects had to decide whether a cue – a faint circular grating – has been present or absent during stimulus presentation) concluded 240 ms post stimulus as the onset of awareness-related activity [80]. A different MEG study yielded similar results, for both auditory and visual conscious perception of novel words [81].

Another approach to determining the onset of conscious processing is by calculating the amount of theta-gamma phase synchrony from brain oscillatory data (see Introduction).

A binocular rivalry experiment using EEG recordings conducted by Doesburg et al. [37] provides supporting evidence for this hypothesis. Doesburg et al. found that gamma-oscillatory networks across the brain, formed and dissolved at the theta frequency band, are time-locked to perceptual switching (they are time-locked to which of the two stimuli the subject is aware of). On a spectral diagram of their results they could identify the times in which the subject was aware of one or the other stimulus, signified by high levels of theta-gamma phase synchronization. The resulting time until one of the stimuli became conscious was 260–380 ms (the temporal distance between the subject being consciously aware of the first and then the second stimulus).The lower time limit is consistent with a previous experiment by the same authors [55], which observed maximal phase synchrony 220–280 ms post stimulus. It is also consistent with the iEEG, EEG and MEG studies described above.

The so called “Visual Awareness Negativity” (VAN), an ERP component defined by the difference between ERPs to conscious versus unconscious stimuli, also fits well into these time ranges, since the part of VAN that is affected by attentional selection occurs at 200–260 ms [82].

Finally, all the results above are to some extent consistent with the time frame of the attentional blink [79], [50]. In attentional blink experiments, two masked visual stimuli are presented in short succession. For short stimulus onset asynchronies, the identification of the first target hinders the detection of the second target (although the second target is easily seen if the temporal distance between the two targets is increased). The worst identification performance of the second stimulus has been observed at delays of about 225 ms between the onsets of the two stimuli [50], which is consistent with the LIDA hypothesis that there can be only one conscious content in one cognitive cycle [8], [16]. This idea is also described by Doesburg et al., who write that after one period of phase synchronization (of the subject being conscious of a stimulus), desynchronization is required before the next period of synchronization; and that during one period of synchronization the subject can be conscious of only one stimulus [37].

It should be pointed out that for determining the time of the conscious broadcast, only the lower limits of the times determined by these experiments are relevant. Cognitive processes after the times indicated by the upper limits in these experimental results presumably include action selection processes (see next section). Therefore, the time range of the conscious broadcast indicated in Figure 6 has been determined by taking into account only the lower limits of these results: the smallest and the greatest lower limit.

Summarizing, consciousness seems to involve large-scale integration of different brain areas through phase coupling, and widespread distribution of sensory information. In simple trials, conscious processing has been estimated to commence C  = 200–280 ms post stimulus (see Figure 6).

### Decision Making/Action Selection

**Figure 7:**
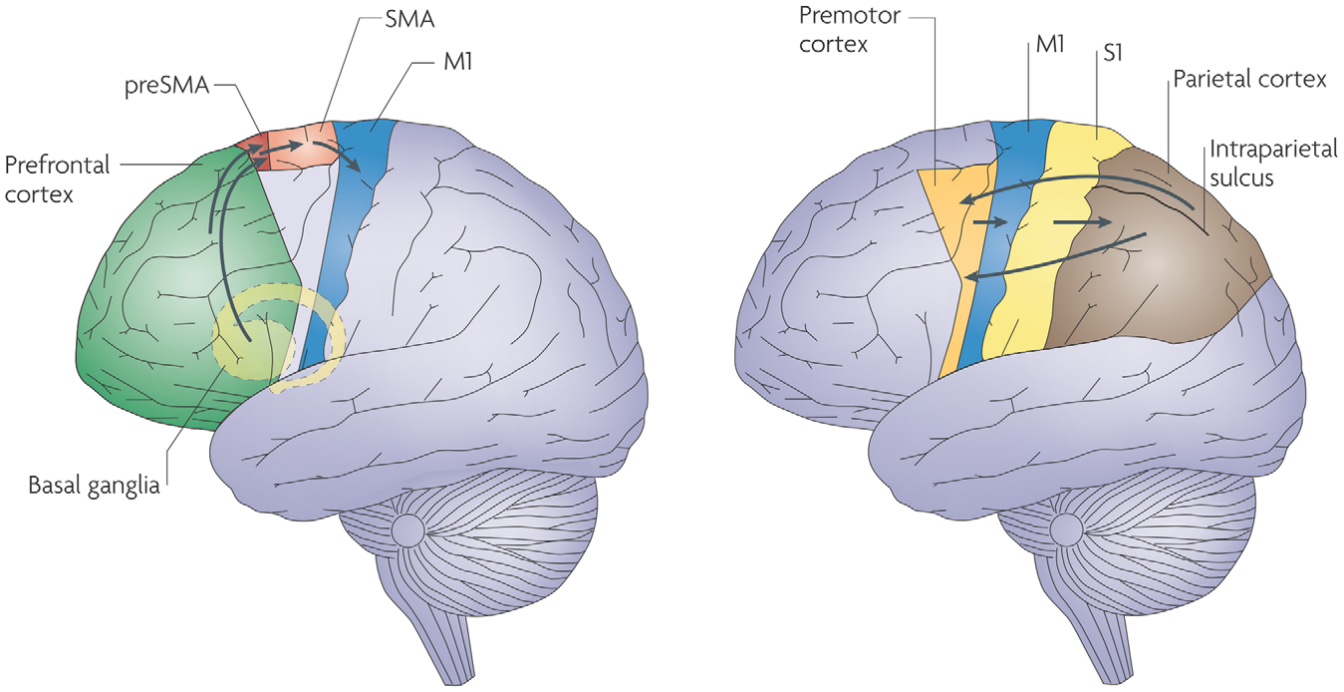
Major brain areas involved in action selection. The left panel shows the brain areas involved when making voluntary actions; the right panel, object-oriented (stimulus driven) actions.

There are several brain circuits involved in action selection, the most relevant being the prefrontal cortex, the pre-supplementary motor area (preSMA), the supplementary motor area (SMA) and the primary motor cortex (M1). Information from the first three areas converges on the primary motor cortex (see Figure 7 - *from* [*83*] *, with permission*), which executes motor commands by transmitting them to the spinal cord and muscles [83]. There can be two classes of inputs to M1, voluntary and stimulus-driven inputs.

The first key input comes into the M1 from the prefrontal cortex by way of the basal ganglia and the preSMA - see the left panel in Figure 7. This circuit is used when making voluntary actions (preSMA activations are stronger for voluntary actions than for stimulus-driven actions).

The second input plays a role in the immediate stimulus-dependent guidance of actions and is projected to M1 from the lateral part of the premotor cortex, which receives its input from the internal representations in the parietal lobe, which in turn are built from information from the sensory cortices (although this circuit also contributes to voluntary behavior) [83] – see the right panel in Figure 7.

The action selection process begins with receiving the conscious broadcast (Figures 5 and 6), and involves two stages:

- the selection of a number of actions that are applicable, depending on the current situation, i.e. the content of the conscious broadcast (represented by the Procedural Memory module in LIDA) and
- the selection of the best available action, i.e. the action that best serves the goals of the agent (represented by the Action Selection module in LIDA).

This separation of action selection into two stages has also been observed in the brain. The brain begins to prepare several actions in parallel while collecting evidence for selecting between them [84], [85]. For example, in visually guided movement, the first stage involves a reciprocally interconnected network of areas in the posterior parietal and caudal frontal cortex, converting sensory information into parameters of potential actions. Each area can represent information that is simultaneously pertinent to several potential actions. There is a competition between these potential actions, corresponding to stage two mentioned above, which is influenced by a variety of brain areas, most importantly the basal ganglia and the prefrontal cortex (for more details see [84]).

There are few experimental results concerning the duration of the action selection process; some of them shall be reviewed below.

In an experiment conducted by Nachev et al. [86], subjects were asked to either follow a specific movement plan or to choose freely between two alternatives in an oculomotor change-of-plan task. After free choice, subjects could be asked to continue their plan or to rapidly change it. Directed trials in which subjects failed to change their planned saccade had latencies 107 ms (median) shorter than trials where the plan change was successful, indicating that the process of selecting a different action took 107 ms.

Taylor et al. [87] have used TMS to interfere with preSMA activity, which disrupted subjects' decision whether they should respond with their left or right hand, if applied in the time window between 180 and 300 ms. Since awareness of a stimulus is a prerequisite of making a conscious decision, the time until the conscious broadcast (200–270 ms, see previous section) can be subtracted from this window, yielding 20–90 ms as the duration of the action selection process.

Philiastides et al. [88] conducted an EEG experiment where subjects had to do a perceptual decision making task, deciding whether there was a face in the shown stimulus (faces in the stimuli had different coherence levels). They found brain activity strongly correlated with the subjects' decision 300 ms post stimulus. They also identified a component at 220 ms the strength of which systematically increased with task difficulty, to which they have assigned the top-down influence of attention (which is consistent with other experiments dealing with attention and consciousness). Subtracting these two times yields an action selection duration of 80 ms.

van Rullen and Thorpe [65] have also conducted an EEG experiment involving a go/no go task with presented visual stimuli (depicting vehicles or animals). Resulting median reaction times were around 350 ms, but they also showed that categorization could be performed above chance after 250 ms (which therefore constitutes the start of the decision process) – implying a duration of ∼100 ms for decision making (action selection).

An MEG experiment by Bauer et al. [89], requiring subjects to perform a simple reaction time task, found high gamma band activity between 200 and 250 ms poststimulus and suggestested a role of this oscillatory activity in crossmodal integration, consistently with the conscious broadcast times described in Section 2.2. In this experiment, average reaction times were 279.1 ms. Subtracting the lower bound of high gamma activity from the reaction time yields 79.1 ms required for both the selection of an action and its execution. It is important to point out that reaction time experiments measuring actual motor responses include both the times of the cognitive cycle sub-processes, and the time for motor execution (which is not included in the described cognitive cycle). The time of the propagation of action potentials, from the motor cortex to evoking hand muscle responses, takes about 20 ms (motor response was evoked 19–24 ms after TMS stimulation of the motor cortex in an experiment by Capaday et al. [90]; which is consistent with the axonal conduction delays of motor neurons [91]). Motor execution can therefore be said to take around 20 ms. This time has to be subtracted from the results of these mechanical reaction time experiments to obtain the cognitive cycle duration. Thus, the action selection part in the experiment of Bauer et al. can be said to take approximately 60 ms.

In the neural action selection circuit described above, we have included not only the selection of an action, but also the selection of the appropriate motor command executed by the motor cortex. These low-level motor commands –information about which muscles or actuators have to be used to implement a specific action – are stored in the Sensory-Motor Memory component in the LIDA model and are chosen after the action selection process. Choosing the exact low-level motor command to use takes a short amount of time in addition to the time taken for action selection. For example, when a person in a restaurant is faced with the decision whether to reach for a glass of wine or a glass of water, his or her brain needs to decide first (select the action) and then choose a low-level motor command (i.e. choose which muscles have to be flexed to reach and grasp the correct glass). The Sensory-Motor Memory has not yet been computationally implemented in LIDA; however, for the simple agents described below, this does not make a difference.

Summarizing, the process of action selection or decision making has been indicated to take 60–110 ms.These times constitute a lower range for the action selection duration in humans, since they were obtained in studies using very simple settings – action selection may very well take longer if the task is more complex. (The 20 ms lower boundary that has been deducted from the Taylor study [87] has been disregarded because it is an outlier compared to the results of other studies).

### Comparison with Psychological Reaction Time

Adding up the durations of the cognitive processes mentioned above yields a total duration of 260–390 ms for a single cognitive cycle (Figure 6). This is on the order of most reaction time experiments from psychology (although slightly longer than most simple reaction time experiments and slightly shorter than most choice task experiments).

The reaction times of young adults has been proposed to be in the range of 190–220 ms [92]. Results from this and other reaction time experiments include the time taken for motor execution, which was not included in our discussion of the cognitive cycle above, and can be said to be around 20 ms (see previous section).The time of the propagation of action potentials, from the motor cortex to evoking hand muscle responses, takes about 20 ms (motor response was evoked 19–24 ms after TMS stimulation of the motor cortex in an experiment by Capaday et al. [90]; which is consistent with the axonal conduction delays of motor neurons [91]). Subtracting this delay, the cognitive cycle duration in these experiments can be inferred to be around 170–200 ms, which is comparable to the lower limit of the cognitive cycle duration described. For choice tasks, reaction times are in the range 356–400 ms if there are two choices [93], which is very close to the upper limit of the proposed cognitive cycle duration.

For more substantial reaction time data, and a more complete survey of reaction time experiments, see [66].

### Comparison with other Cognitive Models

The adaptive control of thought-rational (ACT-R) model, developed mainly by Anderson [64], which is a symbolic cognitive architecture aiming, like LIDA, to explain how the components of the mind work together to produce coherent cognition. Coordination of the ACT-R modules is achieved by a central production system (using production rules). The production system architecture as well as the timing model in ACT-R is very similar to the Executive Process/Integrative Control (EPIC) architecture [65].

Both ACT-R and EPIC processes can be split into the perception, cognition and action sub-processes. ACT-R proposes a duration of 85 ms for the perception process, based on an interpretation of psychological experiments [64]. In EPIC, this time is slightly shorter (50 ms). The time taken by the perception process and the cognition process is 185 ms in ACT-R (150 ms in EPIC), and the time of the action process is 50 ms both in ACT-R and in EPIC. It is important to point out that the action sub-process in ACT-R and EPIC only involves the actual motor execution (unlike our usage of the term, which included obtaining all applicable actions and selecting the appropriate one – these are performed in ACT-R's/EPIC's cognition process).

The Model Human Processor (MHP) proposed by Card, Moran & Newell [94], was an influential cognitive model of human task performance, used to calculate how long it takes to perform a certain task. Card et al. have achieved a good fit of their model to the experimental results from different tasks. Similarly to ACT-R and EPIC, MHP has perception, cognition and action stages. In the original MHP model, perception has been proposed to take 100 ms (with a range of 50–200 ms, depending on the task). The perception and cognition processes together take 170 ms (range: 75–370 ms), and the action process 70 ms (range: 30–100 ms).

**Figure 8:**
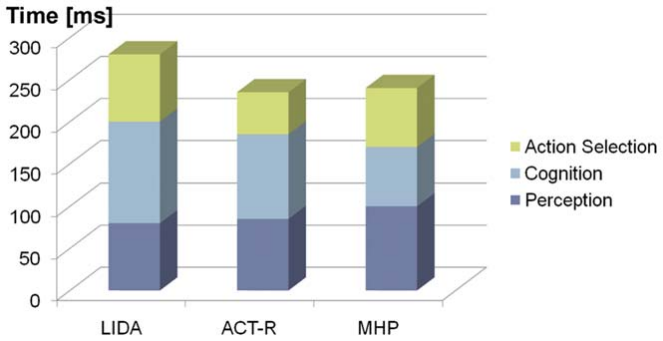
A comparison of the phase timings in LIDA, ACT-R and MHP.

The comparison of these timings with our timing model described above is illustrated by Figure 8. The next two sections will introduce two concrete implementations of agents based on the LIDA model, and compare their performance with human psychological experiments.

### The LRT Agent

A computational framework of the cognitive cycle described in the introduction has been partially implemented [95].

We have developed two autonomous software agents based on this framework, the LIDA Reaction Time (LRT) agent, performing a simple reaction time experiment; and the LIDA Allport Agent, replicating a psychological experiment regarding the continuity of conscious ‘moments’ (see next Section).

**Figure 9:**
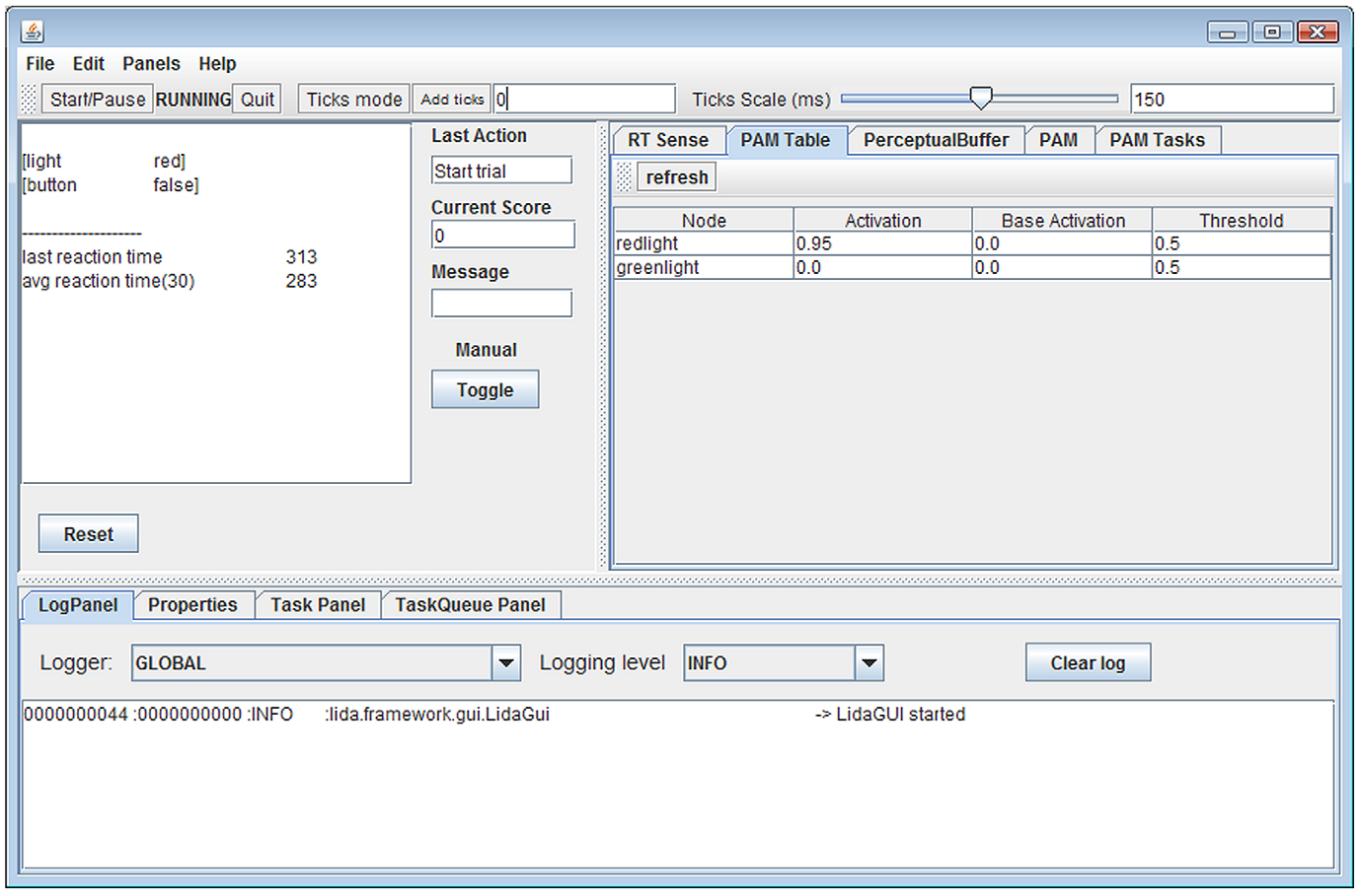
A screenshot of the LIDA Reaction Time Agent. The left top panel contains information about the environment (whether the light is red or green and whether the button is pressed) and statistics about the agent's performance (the last and the average reaction time). The right top panel contains internal information (shown here: the contents of PAM, i.e. the PAM nodes for the red and the green light, and their activations).

The first implementation, the LRT agent, repeatedly performs a reaction time experiment in a simple environment consisting of a light (which can be red or green), and a button (which the agent has to press as quickly as possible when the light turns green). Figure 9 contains a screenshot of the LRT agent. A description of how the LIDA computational model was adjusted for this specific task, as well as a list of parameters tuned to fit the described empirical data, can be found in the Methods section.

**Figure 10:**
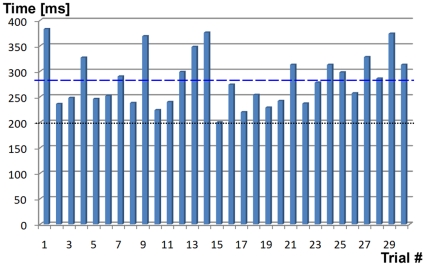
A histogram of the LRT agent's performance at the reaction time task. The blue bars represent the reaction time in single trials. The figure shows n = 30 trials; the average reaction time is 283 ms. The dashed blue line is LRT's average reaction time; the dotted black line represents human reaction time (200 ms, see Decision Making/Action Selection subsection).

Figure 10 shows the LRT agent's performance at the simple reaction time task over 30 trials. As can be seen from this figure, the cognitive cycle durations of the LRT agent (283 ms) are comparable to the cycle durations inferred from the reaction times of adult humans (200 ms according to [92]; see also discussion in the Decision Making/Action Selection subsection), although slightly larger. The main reason for humans being faster at such experiments is the effects of temporal expectation (which has not yet been implemented in LIDA). Humans seem to engage cortical action circuits (inferior parietal and premotor areas) prior to perceiving the stimulus [96], and can thus reduce the time required for action selection after stimulus presentation. Still, the reaction times of humans and of the LRT agent are comparable (the difference is around 40%).

### The LIDA Allport Agent

Allport [58] has conducted an experiment comparing two competing consciousness timing models. Stroud's [57] Discrete Moment Hypothesis, states that consciousness is comprised of distinct and non-overlapping conscious ‘moments’, within which all time-order information is lost, while the Continuous (Traveling) Moment Hypothesis considers conscious ‘moments’ to correspond to continuously moving segments of the incoming sensory information.

Allport's results clearly contradict the strict Discrete Moment Hypothesis. LIDA's discrete consciousness mechanism, however, is consistent with this empirical evidence.

We have successfully replicated Allport's experiment computationally with three goals in mind:

- to show that our discrete consciousness model, based on neuroscientific evidence, does not contradict empirical data - unlike the Discrete Moment Hypothesis (see also the section “LIDA and Consciousness” above),
- to strengthen the claim that LIDA's GWT-based consciousness mechanism models human functional consciousness (note: in an artificial agent we refer to functional consciousness [97], rather than phenomenal consciousness), and
- to substantiate the plausibility of the timing parameters proposed in this paper by showing the similarity of the LIDA Allport agent's behaviour and timing to actual human data.

**Figure 11:**
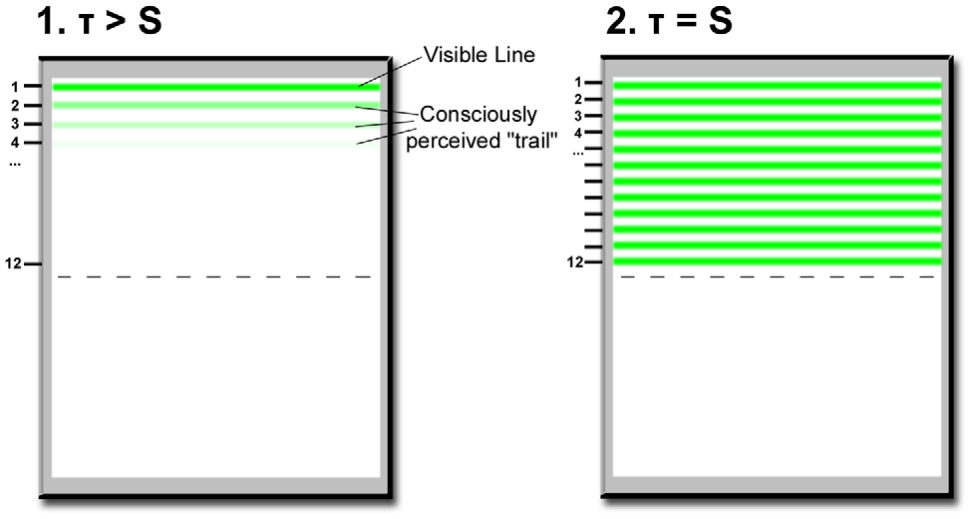
The display and conscious percept in Allport's experiment. τ denotes the total cycle time. At cycle times τ >S, subjects could see multiple lines moving together (left panel). At τ = S, subjects saw all lines simultaneously and perceived no movement (right panel).

In Allport's experiment, subjects were seated in front of an oscilloscope screen, which displayed a single horizontal line, appearing in one of 12 positions on the screen. This line rapidly changed position, moving upward. Upon reaching the topmost position, the screen was left blank for the same duration as the line took while traversing all 12 positions, and then the line appeared again on the bottom position – see Figure 11 (the same visual effect could have been achieved if the line had moved over the whole screen in 24 positions, but with the bottom half of the screen covered). The rate of stepping, and thus the cycle time (τ), was controlled by the subject. At very large cycle times, subjects could see the single line jumping from position to position. Upon decreasing τ, they reported seeing multiple lines, moving together. At a specific cycle time S and below, subjects reported seeing a stationary array of 12 lines flickering in synchrony (see Figure 11).

The subjects had to arrive at the cycle time S, where they did not perceive any movement on the screen. In separate trials subjects first decreased the cycle time from a very high value (slow to fast), and then increased it from a very low value, at which all lines were seen simultaneously (fast to slow). Both times were recorded for each subject. These times were then compared to the predictions of the two hypotheses about consciousness.

**Figure 12:**
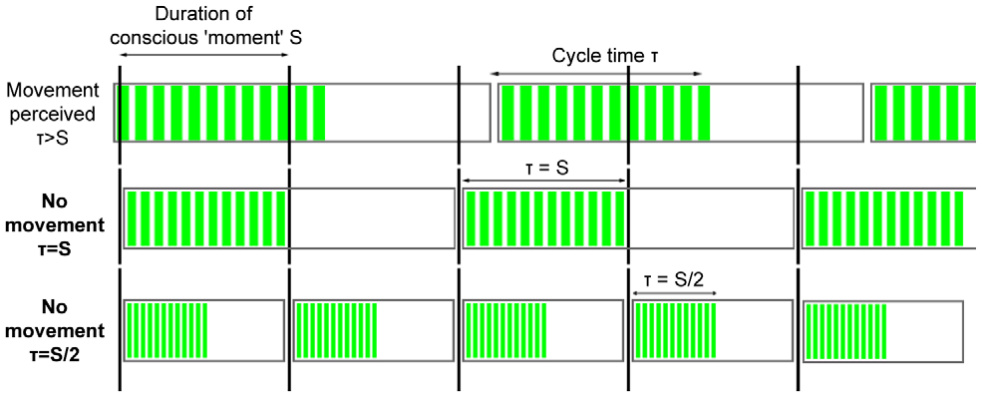
The predictions of Stroud's (1955) Discrete Moment Hypothesis for the Allport experiment. There are two cycle times at which no movement is perceived (τ = S and τ = S/2). Depending on whether the subjects have to increase or decrease the cycle time, they should encounter one or the other. A difference of S/2 is predicted between the two trial types.

According to the Discrete Moment Hypothesis, there are two cycle times at which all 12 lines appear to be on the screen: at τ = S, at which the complete cycle falls within one conscious ‘moment’, and at τ = S/2, at which conscious ‘moments’ containing all lines and no lines alternate (and thus the condition of no movement being perceived is met) – see Figure 12. The cycle time at which subjects will stop, perceiving no movement, will thus be S when decreasing τ, and S/2 when increasing τ. A significant difference between these two conditions is predicted.

The Continuous Moment Hypothesis predicts that successive events are perceived to be simultaneous whenever, and as long as, they fall within the temporal constraints of the conscious ‘moment’. Thus, since the criterion for determining S was not only momentary simultaneity but perpetual absence of perceived movement, there can be only one cycle time S at which this criterion is met (see Figure 12). There should be no difference between trials decreasing or increasing τ.

In [58], twelve subjects performed two versions of this experiment under both conditions:

- the half screen experiment described above, with
  1. decreasing the cycle time until no movement was perceived
  2. increasing the cycle time; and
- the full screen version of the experiment (where the 12 positions were distributed over the entire screen and the line immediately appeared again on the bottom of the screen after reaching the end of the cycle, without delay)
  1. decreasing the cycle time and
  2. increasing the cycle time.

Table 1 displays the resulting cycle times averaged over all subjects (*data from* [*58*]). It is clear that the difference between increasing and decreasing trials is not significant (and certainly not close to S/2), which contradicts Stroud's Discrete Moment Hypothesis.

**Table 1:** Average cycle times at which subjects did not perceive movement in Allport's experiment (n = 12. σ denotes the standard deviation).

*Cycle times τ [ms]* *Human subjects*	1. (decreasing)	2. (increasing)
**A (half screen)**	95,5 (σ = 16,0)	81,4 (σ = 14,6)
**B (full screen)**	86,2 (σ = 12,5)	70,7 (σ = 8,1)

The results from the simulation of these experimental conditions by the LIDA Allport agent are shown in Table 2 below. The data matches Allport's results – there is only one cycle time threshold S at which the agent does not perceive any motion. Despite the high standard deviations of Allport's data, and the as yet imprecise estimates of LIDA's internal parameters, it can be seen from this experiment that the timing data of the Allport agent is comparable to human performance.

**Table 2:** The LIDA Allport agent's cycle times at which the agent did not perceive movement (n = 12).

*Cycle times τ [ms]* *LIDA Allport agent*	1. (decreasing)	2. (increasing)
**A (half screen)**	96	96
**B (full screen)**	84	84

## Methods

### The Implemented Cognitive Cycle

Both agents are based on the almost completely implemented computational LIDA framework, which provides extendable basic implementations for all modules in the LIDA cognitive cycle (Figure 4). These implementations have been extended to allow the agents to perform their respective experiments; and the default timing parameters have been adjusted to fit the empirical evidence described above.

To illustrate how the agents implementations work, we shall describe in this section what happens in each of the modules of the LIDA cognitive cycle outlined in the Introduction; specifically:

1. Sensory Memory
2. Perceptual Associative Memory
  - *(the 2 modules above are part of the* ***Perception*** *phase)*
3. Workspace
4. Attention Codelets
5. Global Workspace
  - *(the 3 modules above are part of the* ***Understanding*** *phase*)
6. Procedural Memory
7. Action Selection
8. Sensory-Motor Memory
  - *(the 3 modules above are part of the* ***ActionSelection*** *phase)*

There are two additional modules in LIDA's cognitive cycle (Transient Episodic Memory and Declarative Memory) which will be omitted here since they are not required in these experiments.

For this simple domain, no visual image processing is necessary. The environment class, which contains and controls the sensory stimulus (and the button), is inspected periodically by the **Sensory Memory** module. The LRT agent's sensory stimulus consists of a single red (or green) light, while the Allport agent's has 12 distinct lines which may or may not be alight.

Simple feature detectors monitor their respective fields in the Sensory Memory, and activate relevant **Perceptual Associative Memory** (PAM) nodes if they find corresponding sensory data. This is comparable to the human visual system, which also makes use of feature detectors – for example, V1 contains neurons that are sensitive to features such as orientation, direction and spatial and temporal frequency, and V4 neurons are sensitive to geometric shapes [67]. In the LRT agent, the single color-sensitive feature detector activates the PAM node representing a red light or a green light, depending on Sensory Memory contents. In the Allport agent, there are 12 feature detectors sensitive to their respective lines, which activate one of the twelve respective PAM nodes upon sensing their line.

Next, the percept (consisting of the identified PAM nodes) is moved into the **Workspace**, which constitutes LIDA's preconscious buffers of working memory. The LRT agent does not use episodic memory, but in the LIDA model, episodic memory contents would be retrieved to the Workspace as well (from the Transient Episodic and Declarative Memories), cued by the percept.

According to Global Workspace Theory, on which LIDA is based, conscious contents reside in a memory capacity that enables access between brain functions that are otherwise separate (see Introduction). In LIDA, this memory capacity is the **Global Workspace**, and its role is enabling the Procedural Memory and the Action Selection access to the most urgent/novel/relevant Workspace contents. These contents are transferred into the Global Workspace by **Attention Codelets** (codelets are special purpose mini-agents implemented as a small piece of code running on a separate thread). These codelets look for their specific concerns in the Workspace and, upon finding it, copy it to the Global Workspace.

An agent is consciously aware^1^ of an object, represented by PAM nodes, the moment these nodes become part of the conscious broadcast (after winning the competition against other contents of the Global Workspace).

Finally, an appropriate action is selected based on the contents in the broadcast. This selection is performed by two components in LIDA. The first component is **Procedural Memory**, from which all behaviours applicable in the current situation are chosen. In the LRT agent, as well as in the Allport agent, there are two possible behaviors (pushing the button, and releasing the button/doing nothing). Note that behaviors could be more complex (they could include many actions) in a more complex domain of application.

The second component is **Action Selection**, in which the action best serving the agent's goal is selected. In the agents described here, this process is trivial – since in all possible states of the environment there is only one applicable action, the Procedural Memory always yields only one action, which only has to be forwarded by the Action Selection component (without competition between actions) to the **Sensory-Motor Memory** for execution. This selected action is then executed in the environment (e.g. the button is pressed). The simple mechanism responsible for this could be called the LRT agent's “actuator”.

### Parameters

As do other computational architectures modeling cognition, LIDA contains a multitude of internal parameters that have to be adjusted for a computational agent acting as subject in the replication of an experiment. Such parameters may include decay rates for various types of memory, a threshold above which a perceptual item becomes part of the current percept, or a parameter that makes action selection more goal-oriented rather than opportunistic. The ultimate goal is a tuned set of internal parameters whose values remain constant when a number of disparate datasets are reproduced. Such a tuned parameter set assures the accuracy and usefulness of the model. Inability to find such a tuned parameter set should warn that the model needs revision. The particular parameters that resist such tuning will point researchers to modules and processes within the model that need revision. This parameter tuning provides a metric for assessing the quality of a cognitive model as a basis for understanding the cognitive processes responsible for the behavior of the agent.

Successfully accomplishing this goal will provide substantial evidence of the accuracy and usefulness of the conceptual cognitive model. Cognitive hypotheses from the model can then be tested by experiments with human subjects to see if their data is predicted by running artificial subjects in the same experimental situations. If so, we will have shown the ability of the theoretical model to predict as well as to explain.

The timing parameters described in this section are a first step in the direction of a well-tuned parameter set for the LIDA model.

Each module in LIDA has a specific task (see module descriptions above) that has to be executed at least once every cognitive cycle. The module tasks are run in a parallel and asynchronous fashion - like the human brain, which does not use sequential information processing, but, rather, local neural circuits which run in parallel.

In the computational framework, all of these module tasks are executed periodically to implement the LIDA cognitive cycle. The execution intervals are governed by ‘ticks’ parameters. These parameters govern in how many ‘ticks’ (simulated milliseconds) a particular task will be executed.

Adjusting these ‘ticks’ parameters, so that the timings of the resulting LIDA cognitive cycle become comparable with the timings of the human action-perception cycle (and, thus, neuroscientifically plausible) was the main purpose of the development of the LRT agent.

The most important parameters resulting from this parameter adjustment are listed in Table 3 below. It is important to point out that the modules corresponding to these parameters do not run in a serial manner - the LIDA model aims for the highest possible asynchrony. The only points in the cognitive cycle where seriality is enforced are the conscious broadcast and the action selection process (the selection of a behavior can only start when the contents of the global workspace become conscious).

**Table 3:** The LRT Agent’s most important timing parameters.

Parameter name	Value [ms]
1. Sensory Memory Ticks	20
2. Feature Detector Ticks	30
3. Attention Codelet Ticks	200
4. NoBroadcastOccurring Trigger	200
5. ProceduralMemory Ticks	110

The first parameter governs how often the contents of the Sensory Memory are updated, i.e. how often the environment is sampled. This would be a domain specific parameter that must be found anew for each LIDA controlled agent implemented.

The second parameter controls how often feature detector codelets are run, detecting features depending on their specialization. Feature detection is very rapid in the LRT agent, as in humans. V1 neuron response latencies start at 30 ms – [98], [99]. Also, a presentation time of 20 ms is required for simple go/no go classification for visual stimuli – [100]. In other experiments, 30 ms was required – [101]. This is also consistent with V1 firing rates, which peak at about 45 spikes per second [102]. In the LRT agent, there are only two Feature Detectors, which detect the color of the light stimulus (one for red and one for green). Upon detecting their corresponding light stimulus, these Feature Detectors pass activation to the corresponding nodes in the Perceptual Associative Memory. If the activation of the updated PAM node exceeds a specific threshold, then a copy of this node is instantiated in the Workspace (LIDA's preconscious working memory).

The next important timing parameter (number 3 in Table 3) governs how often the attention codelets are run. Attention codelets are mini-agents that have the purpose of bringing novel, relevant, urgent, or insistent events to consciousness (i.e. bringing instantiations of their corresponding PAM nodes, or other Workspace structures, to the Global Workspace). Since we have argued that the onset of conscious processing in humans starts at about 200 ms (see Results), this parameter was set to this value. It is important to point out that the conscious broadcast can have multiple triggers. In more complex domains, the broadcast is triggered whenever the cumulative activations of the coalitions built by Structure Building Codelets exceed a specific threshold. The broadcast can also be triggered if a single coalition exceeds another threshold. Both of these thresholds can be interpreted as contents judged novel or important enough being brought to consciousness. Finally, a broadcast is sent automatically if too much time has passed since the last broadcast has commenced. The idea is to allow the conscious processing of less important information in cases when there is no current novel or vitally important content in the Global Workspace (instead of an extended unconscious period that would last until one or more coalitions exceed the activation threshold again). The time at which this trigger is activated, measured from the onset of the last conscious broadcast, is controlled by Parameter 4 (NoBroadCastOccuring Trigger) and was set to 200 ms, the onset of conscious processing in humans, as well.

In the domain of the LRT agent, there is only a single coalition in the global workspace (containing a PAM node representing a red or a green light). A conscious broadcast is automatically triggered whenever the activation of this coalition exceeds a specific threshold. The timing parameters of the Attention Codelet, and those of the perception process, have been chosen in a way that the broadcast happens in the range of 200–280 ms (the range for the onset of consciousness in humans – see the Cognitive Processing and Consciousness Section).

The final parameter (number 5 in Table 3) governs the frequency of the process that leads to the selection of an action. The ‘ProceduralMemory Ticks’ parameter controls how often the set of actions that are applicable in the current situation is retrieved and the actual best action selected. This parameter has been set to 110 ms, the upper limit of the duration of action selection (see Results). As in humans, the duration of the action selection phase will depend on task complexity (especially, on the number of available actions). Since the implementation of the Procedural Memory and the Action Selection components in LIDA are still being worked on, the internal timings of this action selection phase have not yet been determined. But both of these processes have to be rescheduled at intervals longer than the internal processing time they require, to avoid bottlenecks, which is why parameter 5 has been set to the upper limit of the action selection duration described in the Results section. In the current LRT agent implementation, these processes take a very short amount of time; and are rescheduled periodically at intervals indicated by parameter 5in Table 3. For future agents, an improved action selection mechanism based on [103] is in development, which will involve the use of triggers (triggering the selection of the best action, for example, if at least one of the applicable actions has activation above a specific threshold) instead of periodic action selection.

Figure 10 in the results section shows a diagram of the resulting reaction times of 30 trials performed by the LRT agent. For the results of the Allport agent see Table 2 and the previous section. Although setting these parameters and pointing out consistent results does not prove either the cognitive cycle hypotheses or the correctness of our timings, this parameter adjustment has to be done as a prerequisite of building more complex LIDA agents, because the cognitive cycles will have to run at a speed comparable to human cognitive cycles if we expect them to model human cognition (or an aspect thereof). If a number of such LIDA agents, replicating different psychological experiments and thus focusing on different aspects of human cognition, would operate in time frames consistent with the human brain (without readjustments of internal parameters), this would considerably increase the plausibility of the LIDA architecture as a model of human cognition.
